# Epidermal growth factor and aging: A signaling molecule reveals a new eye opening function

**DOI:** 10.18632/aging.100384

**Published:** 2011-09-15

**Authors:** Christopher Rongo

**Affiliations:** The Waksman Institute, Department of Genetics, Rutgers The State University of New Jersey, Piscataway, New Jersey, USA

**Keywords:** EGF, aging, ubiquitin, heat shock protein, C. Elegans

## Abstract

Epidermal Growth Factor (EGF) is known for its role in promoting cell division and cellular differentiation in developing animals, but we know surprising little about what EGF does *in vivo* in mature adult animals. Here I review EGF signaling, emphasizing several recent studies that uncovered an unexpected role for EGF in promoting longevity and healthspan in mature adult *C. elegans*. EGF, acting through phospholipase Cγ and the IP_3_ receptor signaling, maintains pharyngeal and body wall muscle function in aging adults, and delays the accumulation of lipofuscin-enriched aging pigments within intestinal cells. EGF also acts through the Ras/ERK pathway to regulate protein homeostasis by promoting the expression of antioxidant genes, stimulating the activity of the Ubiquitin Proteasome System (UPS), and repressing the expression of small heat shock protein chaperones. The effects of EGF signaling on lifespan are largely independent of Insulin/IGF-like Signaling (IIS), as the effects of EGF signaling mutants on lifespan and heathspan are not affected by mutations in the DAF-2 insulin receptor or the DAF-16 FOXO transcription factor. Nevertheless, these two signal pathways have multiple points of overlap, coordination, and cross regulation. I propose that the IIS and EGF signaling pathways respond to environment and to developmental timing, respectively, so as to coordinate the appropriate physiological strategy that cells use to maintain protein homeostasis.

## The discovery of epidermal growth factor

Many diverse functional roles for Epidermal Growth Factor (EGF) have been uncovered since its discovery by Stanley Cohen more than half a century ago [1]. Cohen had noted that the injection of crude submaxillary gland preparations into newborn mice resulted in increased epidermal growth and keratinization, triggering the premature opening of the eyelid [1, 2]. Using precocious eyelid opening as a bioassay, Cohen isolated and identified EGF as a small secreted protein rich in disulphide bonds [3-6]. Latter experiments showed that EGF could induce mitosis of cultured epidermal cells, promote DNA synthesis, stimulate translation, and increase protein phosphorylation [7-9]. A rush to identify the receptor for EGF and its downstream signaling components ensued.

Our understanding of the function of endogenous EGF signaling *in vivo* was facilitated by developmental genetic studies in several model organisms. In *Drosophila*, there is a single EGF receptor with multiple EGF ligands, and EGF signaling has been implicated in embryonic pattern formation, larval disc proliferation, and multiple cell fate decisions [10-12]. In *C. elegans*, there is a single gene for EGF, called *lin-3*, and a single gene for its receptor, called *let-23*, that were originally identified based on their role in the induction of the nematode vulva from undifferentiated epithelial precursor cells [13, 14]. A single anchor cell situated above the epithelial layer secretes LIN-3/EGF, which acts on the epithelial neighbors nearest to the anchor cell, inducing them to adopt the vulval fate during larval development. Hypomorphic mutations that impair EGF signaling result in a failure of vulval fate specification, resulting in a vulvaless animal that cannot lay eggs (the “bag of worms” phenotype) [15, 16]. EGF is also required for larval survival, as a null mutation in the EGF receptor (EGFR) results in L1 stage larval lethality [17, 18]. Additional functions for EGF include a role in reciprocal signaling from the vulval back to the uterus to coordinate the development of these connected tissues, promotion of male spicule development, and induction of P12.p cell fate in the epithelia [18, 19]. In addition to being a developmental signal, EGF also acts as a physiological signal, promoting ovulatory contractions of the gonad sheath cells and inducing a reversible nervous system quiescence during each larval molt [20, 21].

## EGF signaling can occur through multiple signal transduction pathways

**Figure 1:**
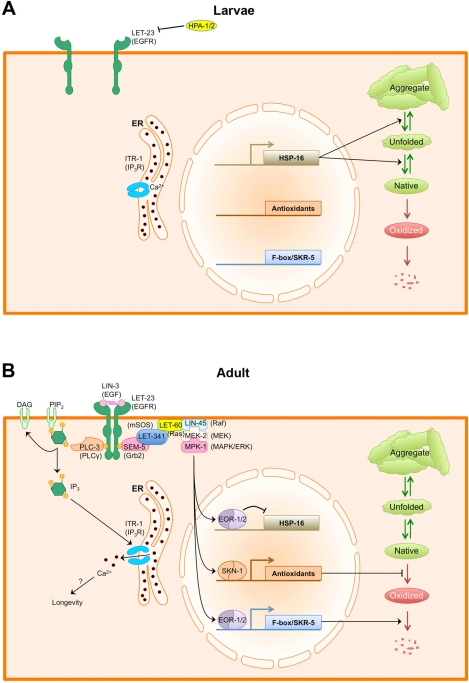
EGF signaling regulates protein and calcium homeostasis in adult *C. elegans*. (**A**) During larval growth, LIN-3/EGF levels in the epithelium are relatively low. Additionally, extracellular HPA-1 and HPA-2 keep EGF signaling repressed by either antagonizing the receptor or the ligand. In the absence of EGF signaling, protein homeostasis is primarily regulated by sHSP chaperones. Native proteins (green elipse) are kept folded and prevented from aggregating by sHSPs. (**B**) As animals enter adulthood, LIN-3/EGF is resynthesized and secreted, activating the LET-23/EGFR. Activated LET-23/EGFR recruits PLC-3/PLCγ, which produces IP_3_ to activate the ITR-1 IP_3_ receptor, resulting in the release of calcium from stores within the ER. How calcium release promotes longevity is not known. Activated LET-23 also recruits the Ras/ERK signaling cascade, which phosphorylates multiple transcription factors, including EOR-1 and SKN-1.SKN-1 activates the transcription of phase 2 antioxidant and detoxification enzymes, which help to minimize protein oxidation. EOR-1 and EOR-2 repress the expression of sHSP genes while activating the expression of multiple F-box proteins and the Cullin1 adaptor SKR-5, promoting global protein turnover. Combined, these changes in the transcriptional profile alter the mechanism for maintaining protein homeostasis from one focused around refolding proteins and preventing aggregation to one focused around preventing the accumulation of oxidized proteins.

A combination of many years of biochemical and genetic studies has elucidated several parallel signal transduction pathways used by EGF and its receptor (Figure 1). Upon EGF binding, the EGFR becomes autophosphorylated, resulting in the recruitment of adaptors like Grb2/SEM-5 and activation of the Ras/ERK signal transduction cascade [22]. In *C. elegans*, LIN-3/EGF activates LET-60/Ras and MPK-1/ERK to promote vulval differentiation through the modulation of multiple transcription factors [23]. EGFR activation also stimulates phospholipase C gamma (PLCγ) [24, 25], resulting in the production of inositol 1,4,5-trisphosphase (IP_3_) and the release of calcium from intracellular stores via the IP_3_ receptor. The *C. elegans* PLCγ and IP_3_ receptor are encoded by PLC-3 and ITR-1, respectively, and *C. elegans* uses this pathway to promote ovulatory contractions in response to LIN-3/EGF [21, 26]. Finally, EGFR activation can in turn promote phosphoinositide 3-kinase (PI3K) activity either directly, with the p85 regulatory subunit of PI3K recognizing the phosphorylated receptor, or indirectly, with p85 interacting through a Grb2/GAB complex. Activated Ras can also activate the PI3K p110 catalytic subunit [27]. Once activated, PI3K converts phosphatidylinositol [4,5]-bisphosphate (PIP_2_) into phosphatidylinositol [3,4,5]-triphosphate (PIP_3_). PIP_3_ in turn binds the pleckstrin homology (PH) domain of Akt, stimulating its kinase activity and promoting the phosphorylation of proteins that regulate cell growth, cell cycle entry, and cellular survival [28]. This includes mammalian target of rapamycin (mTOR), a positive regulator of translation [29]. It is unknown whether *C. elegans* LIN-3/EGF signaling activates PI3K (AAP-1, PDK-1, and AGE-1 in *C. elegans*) or Akt (AKT-1 and AKT-2 in *C. elegans*); however, Insulin/IGF Signaling (IIS) has been implicated in regulating longevity through PI3K and Akt [30].

## EGF implicated in longevity and healthspan

Several recent studies have implicated a novel function for EGF signaling in promoting *C. elegans* longevity. One of these studies was originally prompted from analysis of the IIS pathway, another signaling pathway long implicated in aging. Central to the IIS pathway in *C. elegans* is the DAF-2 insulin receptor, which signals through the AGE-1 PI3K to inhibit longevity [31]. In well-fed animals, DAF-2 becomes activated, triggering the phosphorylation of the DAF-16 FOXO transcription factor by AKT-1, AKT-2, and SGK-1, which prevents it from accumulating in the nucleus [32,36]. By contrast, stress or acute nutrient deprivation depresses IIS activity, releasing DAF-16/FOXO to enter into the nucleus and regulate the transcription of genes involved in dauer formation (a diapause state), metabolism, lipid storage, stress response, and lifespan extension [37,42]. While there is a single DAF-2 insulin receptor, there are forty putative insulin-like ligands in the *C. elegans* genome, as well as multiple insulin receptor-related proteins that are predicted to be secreted molecules [43,45]. Iwasa et al. recently screened these proteins by RNAi-mediated knockdown to identify candidates that might have a role in promoting healthy longevity [46]. Either RNAi-mediated knockdown or deletion mutations for two of these genes, named HPA-1 and HPA-2 for high performance in old age, resulted in increased healthy longevity based on locomotory activity, pharyngeal activity, and age-pigment accumulation in older animals compared to wild-type controls. While HPA-1 and HPA-2 both contain sequences similar to the ligand binding region of the insulin receptor, genetic analysis between *hpa-1*, *hpa-2*,*daf-2*, and *daf-16* mutants indicated that HPA-1 and HPA-2, at least in part, normally function to shorten healthy lifespan by a mechanism that is independent of IIS. Interestingly, HPA-1 and HPA-2 contain leucine-rich domains similar to those of mammalian EGFR-related protein (ERRP), a secreted negative regulator of the EGFR, raising the possibility that HPA-1/2 might inhibit lifespan by antagonizing the LET-23/EGFR [47].

Given its multiple roles in cellular growth and early development, it is not surprising that EGF had not been a major focus of study for a role in lifespan and aging. Indeed, many EGF signaling mutants display developmental defects that preclude analysis of lifespan. To get around this problem, Iwasa et al. examined animals with temperature-sensitive loss of function mutations in the LET-23/EGFR and found that they had a reduced lifespan and diminished health at later developmental time points [46]. Partial loss of function mutations in LIN-3/EGF resulted in a similar longevity phenotype. They also examined animals with a gain of function mutation, *let-23(sa62)*, in the extracellular domain of LET-23/EGFR that results in ligand-independent constitutive activation of the receptor [48]. Gain of function *let-23* mutants showed increased longevity, delayed sarcopenia and age pigment accumulation, and healthier locomotion behaviors later in life compared to wild-type controls. In contrast to the effects of IIS pathway mutations, the effects of EGF signaling pathway mutations on lifespan were not additive with the effects of RNAi knockdown on HPA-1 and HPA-2, consistent with HPA-1/2 functioning in the EGF signaling pathway, most likely by antagonizing EGFR function or EGF/EGFR interaction.

Iwasa et al. examined mutations in the different signaling pathways downstream of LET-23/EGFR [46]. RNAi knockdown of LET-60/Ras and MPK-1/ERK did not block the extended lifespan observed in *let-23(sa62)* gain of function mutants. By contrast, either RNAi knockdown or mutations in PLC-3/PLCγ or the ITR-1/IP_3_ receptor blocked the increase in healthy aging observed in *let-23(sa62)* mutants. Loss of function and gain of function mutations in ITR-1 resulted in reduced and enhanced lifespan, respectively, even in the absence of *let-23(sa62)* mutations, indicating that EGF signaling promotes healthy aging in *C. elegans* through activation of PLCγ and the IP_3_ receptor, presumably triggering calcium release from internal stores. It remains unclear how accelerated release from internal calcium stores could promote longevity, although one possibility could be that alterations in ovulation, which are regulated by EGF/PLCγ/IP_3_ signaling in the *C. elegans* gonad, contribute to the observed changes in healthy lifespan [49]. Alternatively, elevated cytosolic calcium might activate beneficial calcium-dependent pathways that promote longevity.

Other branches of the EGF signal transduction pathway besides just the PLCγ/IP_3_ pathway contribute to aging. Recently, Okuyama et al. tested for a role for Ras/ERK signaling in lifespan regulation [50]. To get around the problem of developmental defects, they performed RNAi knockdown of LIN-45/RAF, MEK-2/MEK, and MPK-1/ERK after animals had reached adulthood. Knockdown of these genes, as well as treatment with the MEK inhibitor U0126, resulted in a shorter lifespan. By contrast, knockdown of LIP-1, a phosphatase that normally antagonizes MPK-1/ERK, resulted in lifespan extension [51]. One of the phosphorylation substrates of mammalian ERK is Nrf2, a transcription factor that mediates the antioxidant response during oxidative stress [52]. In *C. elegans*, Nrf2 is encoded by SKN-1, which is required for normal lifespan and stress resistance [53, 54]. MPK-1/ERK phosphorylates SKN-1, promoting its activation and nuclear accumulation, and knockdown of MPK-1/ERK by RNAi does not result in additional lifespan reduction in the absence of functional SKN-1, suggesting that Ras/ERK signaling can promote lifespan extension, at least in part, through activation of SKN-1 and the antioxidant response [50].

## EGF regulates protein homeostasis via the Ubiquitin Proteasome System

Activation of SKN-1 is not the only way by which EGF signaling through Ras/ERK affects lifespan. Indeed, a role for EGF in longevity was also recently demonstrated by Liu et al., using a completely different approach [55]. These researchers were examining protein homeostasis regulation by the Ubiquitin Proteasome System (UPS) in *C. elegans* using an Ub^G76V^-GFP chimeric reporter for UPS activity. Chimeric Ub^G76V^-GFP protein contains an amino-terminal ubiquitin fused in frame with GFP, but with a mutation at glycine 76 that prevents the cotranslational cleavage that would otherwise release ubiquitin from GFP after synthesis [56-58]. The resulting uncleaved Ub^G76V^-GFP protein mimics a monoubiquitinated GFP and is an efficient, non-specific substrate for additional polyubiquitination and proteolysis by the 26S proteasome. Ub^G76V^-GFP essentially acts as an inverse reporter for UPS activity, yielding high GFP fluorescence when UPS activity is low and vice versa. Liu et al. used different cell-type specific promoters to express Ub^G76V^-GFP in different *C. elegans* tissues. They noticed that Ub^G76V^-GFP levels in larvae remained relatively high in epithelia, but that epithelial Ub^G76V^-GFP was rapidly degraded as animals matured into fertile adults, suggesting that the steady state levels of UPS activity are low during early development, but become enhanced at a specific point in adult maturation. To identify the biological signal that triggers this augmentation in UPS activity in adulthood, they undertook a candidate gene approach looking at known genes that had been previously implicated in regulating protein turnover. A role for fibroblast growth factor (FGF) and Ras/ERK signaling in protein turnover had previously been demonstrated in *C. elegans* body wall muscle [59]. Liu et al. found that whereas Ras/ERK signaling was required for Ub^G76V^-GFP turnover in adult epithelia, FGF was not required. Reasoning that the FGF ligand might be specific for muscle, and that different tissues might use different signaling ligands to regulate UPS activity, they examined EGF signaling mutants, including *lin-3* and *let-23*. Loss of either LIN-3/EGF or LET-23/EGFR prevented the rapid degradation of Ub^G76V^-GFP as animals entered adulthood. By contrast, the *let-23(sa62)* gain of function mutation in the EGFR resulted in precocious turnover of Ub^G76V^-GFP during larval development. Thus, EGF signaling through the Ras/ERK pathway, but not through the PLCγ/IP_3_ pathway, was directing protein turnover in adult epithelia.

Using this same candidate gene approach, Liu et al. also identified components of the Ubiquitin Fusion Degradation (UFD) machinery as being required for Ub^G76V^-GFP turnover [55]. The UFD is a collection of E3 ubiquitin ligases and E4 polyubiquitin extension enzymes that recognize protein substrates attached with a small chain (1-3 units) of ubiquitin and then catalyze the addition of more ubiquitin molecules, resulting in an extended polyubiquitin chain that targets the attached substrate protein for degradation [57, 60-62]. Interestingly, Liu et al. found that mutants for multiple UFD component genes had a decreased lifespan, similar to the lifespan of EGF mutants revealed by Iwasa et al. Importantly, mutations in these UFD genes could suppress both the lifespan extension and the premature UPS activation observed in *let-23* gain of function mutations. These findings suggested that EGF pro-motes longevity by tuning the level of UPS activity at different stages of development, presumably having a significant impact on protein homeostasis. The finding that Ras/ERK signal transduction is required for EGF to activate the UPS strongly suggested that the connection between EGF signaling and UPS activity would involve transcriptional changes.

A number of transcription factor targets of EGF signaling are known in *C. elegans*. Whereas the transcriptional targets that mediate vulval differentiation do not appear to be required for regulated UPS activity, the transcription factors EOR-1 and EOR-2 are essential for the activation of the UPS in adults [55]. Indeed, mutations in *eor-1* completely blocked the accelerated UPS turnover observed even in the gain of function *let-23* mutants. EOR-1 is similar to PLZF, a transcription factor implicated in acute promyelocytic leukemia [63, 64]. Microarray analysis yielded an expression profile for EOR-1-regulated genes, including multiple target genes involved in the UPS, fat metabolism, and the heat shock chaperone response, as well as several genes with previously identified roles in regulating longevity (several are targets of DAF-16/FOXO).

Interestingly, EGF signaling through EOR-1 results in the downregulation of small Heat Shock Proteins (sHSPs) of the Hsp16 family [55]. Hsp16 chaperones act in the cytosol to prevent the aggregation of misfolded and damaged proteins, and appear capable of reversing protein aggregation [65-67]. As a consequence, mutants defective in EGF/EOR-1 signaling have elevated Hsp16 chaperone levels and thus accumulate aggregates of metastable proteins at a slower rate than that of wild type [55]. By contrast, mutants with enhanced EGF signaling, like *let-23(sa62)* mutants, have reduced Hsp16 levels and are more susceptible to protein aggregation. Thus, EGF signaling affects protein homeostasis by modulating the cellular anti-aggregation response.

Concurrently, EGF signaling through EOR-1 also results in the upregulation of SKR-5, a Skp1-like adaptor protein [55]. Skp1 adaptors act to recruit different F-box substrate recognition proteins to Cullin1 scaffolding molecules, facilitating the formation of hundreds of different E3 ubiquitin ligases [68-71]. The *C. elegans* genome contains a single Cullin1 gene and 21 Skp1-like adaptors [72]. Perhaps the upregulation of SKR-5 by EGF/EOR-1 signaling results in the activation of multiple different F-box/E3 ubiquitin ligase complexes? Consistent with this possibility, loss of function mutations in *skr-5* prevent the activation of the UPS as animals enter adulthood and have an accelerated aging defect similar to that of mutants for UFD complex components [55]. Mutations in *skr-5* also block the precocious activation of the UPS and the increased longevity observed in *let-23(sa62)* mutants. Whereas upregulation of the SKR-5 adaptor is probably not the entire explanation for the activation of the UPS in adults, it does suggest that by regulating the expression of specific adaptors, cells can change whole batteries of different E3 ligases, and hence the stabilities of their different target proteins. In the case of EGF signaling, this mechanism might be regulating aging by simply increasing the surveillance of oxidized proteins. Alternatively, it could be regulating other signaling pathways involved in aging. For example, several E3 ubiquitin ligases have been implicated in aging through their regulation of DAF-16/FOXO or PHA-4/FOXA [73-79].

## EGF signals a change in strategy for maintaining protein homeostasis

**Figure 2:**
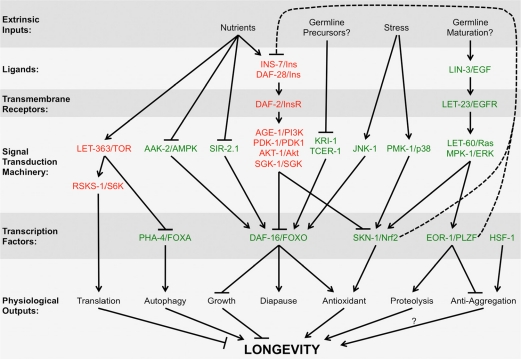
Multiple pathways interact with the EGF pathway to regulate longevity Different steps from multiple signal transduction pathways known to regulate longevity are shown on each row, including extrinsic inputs into the pathway (e.g., nutrient status, environmental stress, et cetera), extracellular ligands, transmembrane receptors, intracellular signaling cascades, transcription factors, and ultimate physiological outputs. Arrows indicate positive “stimulatory” genetic interactions, whereas T-bars indicate negative “repressive” genetic interactions. Dotted lines indicate feedbacks loops. Red letters indicate genes whose wild-type function ultimately acts to shorten lifespan, whereas green letters indicate genes whose wild-type function ultimately acts to enhance lifespan. Not all interactions should be considered of equal weight (e.g., different signaling pathways are activated depending on the timing and degree of nutrient deprivation). Additional reviews describe these other signaling pathways in more detail [30, 81-91].

Taken together, it would appear that EGF is used as a signal not only for tissue morphogenesis during development, but for regulating tissue physiology as well. Initially, nematodes use LIN-3/EGF to induce epithelia morphogenesis during larval development. As nematodes enter adulthood, their epithelia resynthesize LIN-3/EGF, perhaps as an autocrine signal, to trigger the activation of multiple signal transduction pathways, altering calcium homeostasis, translation, protein folding and anti-aggregation, and UPS activity and protein turnover (Figure 2). This represents a strategic, genetically programmed switch in cellular physiology as animals mature into adulthood, with timing that closely parallels the final maturation of the germline, suggesting that these two events might be coupled. Given the close timing to germline maturity, its reasonable to speculate that this EGF-triggered switch in physiology might have evolved to maximize organismal health at the period of peak fecundity. If this is the case, then the impact of EGF signaling on aging might simply be a lucky consequence of optimized fitness during young adulthood. By maximizing organismal health during the period of fecundity, the animal is able to ward off physiological decline associated with aging even long after it has depleted its germ cells and fertility no longer matters, with the added benefit of extended longevity, at least under laboratory conditions.

**Figure 3:**
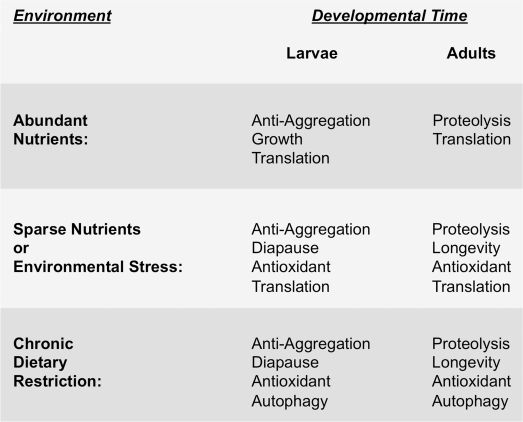
Environment and developmental timing to-gether specify the physiological strategy cells use to maintain protein homeostasis The left hand column indicates different environmental conditions that nematodes encounter, ranging from well fed (i.e., abundant nutrients), to acute or sparse nutrient availability, to more severe and chronic dietary restriction. The right hand columns show the resulting predominant physiological state that occurs depending on the stage in development (i.e., larvae versus adulthood) at which the animal encounters the indicated environmental conditions. We hypothesize that signaling pathways like the EGF pathway and IIS pathway respond to environmental conditions and to developmental timing so as to coordinate the appropriate physiological response.

A number of important questions remain. What triggers the reactivation of LIN-3/EGF expression in adults? Is EGF signaling and UPS upregulation coupled to the maturation of the germline? Or are other developmental timing mechanisms involved? What is it about calcium release from internal stores that results in increased longevity? Is the turnover of all proteins affected by EGF signaling, or just specific subsets? What are the different F-box proteins that interact with SKR-5 as opposed to the other Skp1-like adaptors? Finally, how do the EGF and IIS pathways interact? There are common transcriptional targets between EOR-1 and DAF-16, including SKR-5 [41, 55], and EOR-1 upregulates INS-7, a ligand for DAF-2 with a known role in lifespan [80]. In addition, both IIS and EGF signaling converge on the transcription factor SKN-1, and SKN-1 in turn feeds back on IIS signaling through its regulation of INS-39 and DAF-28 [50]. The combined pathways, with IIS responding to nutritional status and EGF signaling responding to developmental timing, together create the physiological state most suited for surviving the environment at that stage in life (Figure 3). Future efforts towards understanding how these pathways interact will have important implications for understanding not only the aging process itself, but cancer, neurodegenerative disorders, and other diseases that are associated with aging.
